# An indirect estimation of the population size of students with high-risk behaviors in select universities of medical sciences: A network scale-up study

**DOI:** 10.1371/journal.pone.0195364

**Published:** 2018-05-08

**Authors:** Homeira Sajjadi, Zahra Jorjoran Shushtari, Mohsen Shati, Yahya Salimi, Masoomeh Dejman, Meroe Vameghi, Salahedin Karimi, Zohreh Mahmoodi

**Affiliations:** 1 Social Determinants of Health Research Center, University of Social Welfare and Rehabilitation Sciences, Tehran, Iran; 2 Department of Aging, University of Social Welfare and Rehabilitation Sciences, Tehran, Iran; 3 Social Development and Health Promotion Research Center, Kermanshah University of Medical Sciences, Kermanshah, Iran; 4 Department of Epidemiology, School of Public Health, Kermanshah University of Medical Sciences, Kermanshah, Iran; 5 Department of Mental Health, Johns Hopkins Bloomberg School of Public Health, Baltimore, United States of America; 6 Social Welfare Management Research Center, University of Social Welfare and Rehabilitation Sciences, Tehran, Iran; 7 Social Determinants of Health Research Center, Tabriz University of Medical Sciences, Tabriz, Iran; 8 Social Determinants of Health Research Center, Alborz University of Medical Sciences, Karaj, Iran; Institut Català de Paleoecologia Humana i Evolució Social (IPHES), SPAIN

## Abstract

**Background:**

Network scale-up is one of the most important indirect methods of estimating the size of clandestine populations and people with high-risk behaviors. The present study is an indirect estimation of the population size of students with high-risk behaviors in select universities of medical sciences.

**Methods:**

A total of 801 students from two University of Medical Sciences at Tehran and Alborz University of Medical Sciences were selected through convenience sampling. Six subgroups of high-risk behaviors were examined in the study, including Tramadol use, cannabis use, opium use, alcohol consumption, extramarital heterosexual intercourse, and heterosexual intercourse in return for money. To estimate the social network size in the study population, each participant was asked to name their close student friends from the two select universities. Data were collected using a checklist designed for this purpose.

**Results:**

The participants’ mean number of close friends from the selected medical universities was C = 8.14 (CI: 7.54–8.75). Within these social networks, friends with extramarital heterosexual intercourse (5.53%) and friends who consumed alcohol (4.92%) had the highest frequency, and friends who used opium (0.33%) had the lowest frequency. The variables of age, gender, marital status, type of residence and academic degree were significantly related to the likelihood of having close friends with certain high-risk behaviors (P<0.001).

**Conclusion:**

According to the results obtained, alcohol consumption and extramarital heterosexual intercourse are very common among students. Special HIV prevention programs are therefore necessary for this age group.

## Introduction

One of the most important public health problems in Iran in the recent decade has been the growing trend of high-risk behaviors, especially among young people. The youth population is a vital subgroup of the society that comprises about one-third of the total population in Iran. Whenever the country is in a state of transition and is undergoing socioeconomic changes, this subgroup of the population happens to be most exposed to changes in behavioral patterns and social relations. High-risk behaviors such as drug use and alcohol consumption and extramarital sex are increasing among young people in Iran, thereby exposing this group of the population to the risk of various diseases such as HIV [1, 2]. According to the latest national report on the progress of HIV in 2015, 19.5% of the 20-29-year-old population has extramarital sexual intercourse [3]. The results of studies on smoking and drug use behaviors suggest a relatively high frequency for these behaviors among young people [4–7]. In a study conducted on university students in Rasht, the prevalence of smoking was reported as 24.13%, alcohol consumption as 10.5%, ecstasy consumption as 7.25% and opium use as 4.87% [8].

A few studies have estimated the prevalence of high-risk behaviors among students; however no accurate statistics are available on the frequency of high-risk behaviors among the youth, especially the young students.

The existing studies have used indirect methods to estimate the prevalence of these behaviors, while high-risk behaviors, e.g. the use of narcotics and stimulants or extramarital sexual intercourse, are illegal in Iran and carry a social stigma and are looked on with great prejudice.

People with high-risk behaviors engage in these types of activities in a clandestine manner and therefore make up an inaccessible part of the population. In cases where they are accessible and agree to participate in studies, they often refrain from truthfully discussing their illegal high-risk behaviors in the attempt to avoid social stigma. The existing estimations made through direct methods are therefore expected to suffer from the social desirability and the undercount bias[2, 9, 10].

Network Scale-Up (NSU) is one of the most important indirect methods of estimating the size of clandestine populations and people with high-risk behaviors that requires no direct contact with people. In this method, a random sample is used from the general population to estimate the size of the social networks in that population and the number of people belonging to the intended subgroups in these networks. This method can be used to simultaneously estimate the population of several groups [11–13].

Evidence suggests that indirect methods such as NSU are better able than direct methods to provide an accurate estimation of the population size of clandestine groups with high-risk behaviors [14–18]. Identifying the population size of young people with high-risk behaviors is the missing link of planning and policy-making for preventive interventions targeting this important subgroup of the active and productive part of the population.

Accurate data are required on the size of the student population with high-risk behaviors for better decision-making, the timely planning and implementation of interventions, the evaluation of the existing health programs and policies and optimal resource allocation [2, 10].

The present study was conducted to estimate the population size of students with high-risk behaviors at Tehran University of Medical Sciences and Alborz University of Medical Sciences.

## Materials and methods

### Study design and sample size

The present cross-sectional study was conducted between spring and winter 2015 on 801 students in three medical universities at Tehran and Alborz using NSU. After obtaining the necessary permissions for conducting the study, a probability proportional to size sampling was used for participant selection. In the next step, because of the study questionnaire included sensitive items on the students’ high-risk behaviors, a sampling method capable of yielding the highest response rate had to be chosen. The researchers therefore visited the select university campuses and used convenience sampling to select their participants. Since it was impossible to obtain a full list of the students in each university, the sample studied was not randomly selected; rather, convenience sampling was used.

The study inclusion criteria consisted of having been a student for at least one year in two universities at Tehran or Alborz medical university and being of Iranian nationality. The first criterion was added to the list because a minimum of one year was needed for the students to be able to come up with their social network in the university. Due to the large population size and its dispersion, the researchers trained five interviewers for the project. All the data about the high-risk behaviors of the students’ close friends were obtained in an anonymous form without including any name and personal information from the students. The interviewers briefed the participants on the study objectives and ensured them of the confidentiality of their information and it not being used against them anywhere. The willing candidates then submitted their informed written consent.

### Data collection and instruments

Data were collected using a checklist for this purpose that contained two main sections: first, a section on participants’ demographic details (age, gender, marital status, academic degree, type of residence and marital status), the size of their social networks and the number of their friends who fell in each of the six subgroups under study (Tramadol users, cannabis users, opium users, alcohol consumers, friends engaging in extramarital heterosexual intercourse and friends engaging in heterosexual intercourse in return for money); second, a section inquiring about participants and the certainty or uncertainty of participants’ knowledge of these people.

To estimate the size of participants' social networks, they were asked to name their close student friends in the two selected medical universities.

The size of participants' social network (C) was defined as the number of close student friends each participant had from one of the two medical universities, and "close friend" had a specific meaning and encompassed a defined span of time and place in this study and included student friends from one of the two universities whom the participant had known for at least six months and with whom they had eaten at least one meal together and spent two hours hanging out over the past two weeks [19]. Members of the six subgroups were defined as close student friends who had used narcotics or stimulants and had extramarital heterosexual intercourse at least once in the past year.

### Data management and analysis

The bivariate analysis was used to assess the demographic variables' relationship with the size of participants' social networks and their likelihood of having at least one close friend with high-risk behaviors. Variables with P<0.2 were included in the multiple linear regression analysis. In the population size estimation equation using the NSU, *e* denotes the population size of the target group, *m* shows the frequency of the target group in participants' social networks and *T* denotes the total population size. The frequency calculation method with a 95% confidence interval was used to estimate the population size of the target groups. The Visibility Factor (VF) were calculated to correct the estimated sizes. The VF is the percentage of alters who are aware of the high-risk behaviors of the Egos. To this end, 30 students from the target group with high-risk behaviors (each subject was referred to as an Ego) entered the study after they were briefed on the study objectives and gave their verbal consents [20, 21]. To assess the VF, each participating student with high-risk behaviors (Ego) was asked "How many of the close friends you mentioned know about your high-risk behaviors, such as your use of alcohol, opium, cannabis and tramadol or your extramarital heterosexual intercourse and engagement in heterosexual intercourse in return for money or goods?". The VF is obtained by dividing the number of informed alters (a_j_) by the total number of alters (t_j_):

$$VF=\frac{\sum_{j}aj}{\sum_{j}tj}$$

The data obtained in the study were analyzed in SPSS-17 and Excel-2010.

## Ethical considerations

The research project was approved by the ethics committee of the University of Welfare and Rehabilitation Sciences in Tehran (ID: Ir.uswr.rec.1393.95) and Alborz University of Medical Sciences (ID: Ir.abzums.ac.ir.rec.1393.2).

## Results

The mean (SD) age of the participants was 22.41(3.14) years. The majority of them were male (61.8%) and single (92.4%) and almost half lived in the dormitory (Table 1).

**Table 1:** Participants’ demographic variables (n = 801).

Variable		Frequency	Percentage
Age	20<	253	6.31
	20–24	381	47.6
	25>	167	20.8
Gender	Male	495	61.8
	Female	306	38.2
Marital Status	Single	741	92.5
	Married	60	7.5
	Divorced	0	0
Type of Residence	Dormitory	442	55.2
	Separate Home	9	1.1
	Shared Home	19	2.4
	Family Home	329	41.1
Academic Degree	Bachelor's	361	45.1
	Master's	129	16.1
	General Practice	269	33.6
	PhD	40	4.99

Their mean number of close student friends from the medical universities was C = 8.14 (CI: 7.54–8.75). According to the results of the multiple linear regression analysis, gender, academic degree and type of residence showed a significant relationship with the mean size of participants' social networks once the effect of the other variables was kept constant. The female students had significantly smaller social networks than the male students (P<0.001). The postgraduate students and those who lived with their families also had significantly smaller social networks compared to students from other degrees and those who lived in the dormitory (P<0.01; Table 2).

**Table 2:** The relationship between participants’ demographic variables and their social network size.

Variable		Mean (SD)	P-Value	P-Value
Age	20<	8.45(9.37)	-	-
	20–24	9.02(9.38)	<0.01	0.95
	25>	5.68(4.59)	0.417	0.09
Gender	Male	9.28(8.9)	-	-
	Female	6.30(7.85)	<0.001	<0.001
Marital Status	Single	8.40(8.90)	-	-
	Married	4.98(4.33)	<0.01	0.26
	Divorced	-	-	-
Type of Residence	Dormitory	9.14(10.46)	-	-
	Family Home	6.97(5.67)	<0.01	<0.01
	Separate Home	4.67(3.80)	0.12	0.11
	Shared Home	6.48(3.06)	0.25	0.24
Academic Degree	Bachelor's	9.08(9.51)	-	-
	Master's	5.91(5.77)	<0.001	<0.05
	General Practice	8.41(8.90)	0.33	0.70
	PhD	5.05(4.40)	<0.01	0.43

The highest frequency of friends in participants' social networks pertained to close friends with extramarital heterosexual intercourse (5.53%) and the alcohol consumers (4.92%) and the lowest frequency to close friends who used opium (0.33%). Table 3 presents the frequency of close friends with high-risk behaviors in participants' social networks.

**Table 3:** The frequency of having at least one close friend with high-risk behaviors in participants' social networks (C = 6524).

High-Risk Behavior	Frequency	Percentage
Tramadol Use	92	1.41
Alcohol Consumption	321	4.92
Opium Use	22	0.33
Cannabis Use	40	0.61
Extramarital Heterosexual Intercourse	361	5.53
Heterosexual Intercourse in Return for Money	131	2

According to the results obtained, the likelihood of having close friends with certain high-risk behaviors was significantly related to the variables of age, gender, marital status, type of residence and academic degree. Compared to men, women had a smaller chance of having at least one close friend who used tramadol, had extramarital sex or engaged in extramarital heterosexual intercourse in return for money or no money (P<0.001). According to the multivariate logistic regression analysis, the likelihood of having a close friend who used tramadol (P<0.05), consumed alcohol (P<0.01) and had extramarital heterosexual intercourse (P<0.05) was significantly higher in the social network of the students who shared homes with other students compared to those who lived in the dormitory. Compared to the single students, the married students had a smaller chance of having at least one close friend who consumed alcohol and had extramarital heterosexual intercourse (P<0.05; Table 4).

**Table 4:** The relationship between the demographic variables and the likelihood of having at least one close friend with high-risk behaviors (n = 801).

Variable		Tramadol Use				Alcohol Consumption				Opium Use				Cannabis Use				Extramarital Heterosexual Intercourse				Heterosexual Intercourse in Return			
		Crude OR	P-value	Adjusted OR	P-value	Crude OR	P-value	Adjusted OR	P-value	Crude OR	P-value	Adjusted OR	P-value	Crude OR	P-value	Adjusted OR	P-value	Crude OR	P-value	Adjusted OR	P-value	Crude OR	P-value	Adjusted OR	P-value
Age	<25	-	-	-	-	-	-	-	-	-	-	-	-	-	-	-	-	-	-	-	-	-	-	-	-
	20–24	2.11	<0.05	1.48	0.19	1.35	0.074	1.46	<0.05	0.065	0.37	0.57	0.25	2.36	0.05	2.94	<0.05	1.75	<0.01	1.40	0.05	0.46	<0.001	0.88	0.62
	>25	1.88	0.06	1.54	0.33	1.36	0.13	1.48	0.15	0.67	0.51	0.52	0.42	2.0	0.18	3.18	0.05	2.14	<0.001	1.93	<0.05	0.51	<0.05	0.85	0.75
Gender	Male	-	-	-	-	-	-	-	-	-	-	-	-	-	-	-	-	-	-	-	-	-	-	-	-
	Female	0.13	<0.001	0.15	<0.001	1.15	0.37	1.19	<0.02	1.63	0.25	1.81	0.19	0.97	0.92	0.98	0.94	0.42	<0.001	0.45	<0.001	25.6	<0.001	23.7	<0.001
Marital Status	Single	-	-	-	-	-	-	-	-	-	-	-	-	-	-	-	-	-	-	-	-	-	-	-	-
	Married	0.39	0.11	0.30	0.08	0.52	<0.05	0.46	<0.05	1.0	0.99	1.0	0.99	0.64	0.54	0.52	0.42	0.64	0.11	0.50	<0.05	0.66	0.31	0.61	0.35
Type of Residence	Dormitory	-	-	-	-	-	-	-	-	-	-	-	-	-	-	-	-	-	-	-	-	-	-	-	-
	Family Home	0.63	0.06	0.76	0.3	1.40	<0.05	1.40	<0.05	0.39	0.06	0.47	0.16	1.69	0.11	1.56	0.21	0.84	0.23	1.06	0.73	3.63	<0.001	3.07	<0.001
	Separate Home	0.88	0.90	0.89	0.9	2.29	0.22	1.60	0.492	1.0	0.99	1.0	0.99	1.0	0.99	1.0	0.99	1.47	0.57	1.74	0.43	2.89	0.19	1.69	0.59
	Shared Home	6.36	<0.001	4.25	<0.05	9.75	<0.001	7.06	<0.01	1.0	0.99	1.0	0.99	1.0	0.99	1.0	0.99	4.41	<0.01	3.59	<0.05	1.19	0.82	2.0	0.45
Academic Degree	Bachelor's	-	-	-	-	-	-	-	-	-	-	-	-	-	-	-	-	-	-	-	-	-	-	-	-
	Master's	0.83	0.57	0.62	0.24	1.64	<0.05	1.52	0.09	1.31	0.59	2.02	0.28	1.56	0.39	1.27	0.68	1.59	<0.05	1.21	0.45	1.61	0.09	2.24	<0.05
	General Practice	1.17	0.53	1.20	0.51	2.03	<0.001	1.78	<0.01	0.30	0.06	0.35	0.11	2.85	<0.01	2.99	<0.01	0.95	0.77	0.91	0.58	2.55	<0.001	2.17	<0.01
	PhD	1.43	0.45	1.49	0.56	1.0	0.99	1.03	0.95	1.0	0.99	1.0	0.99	0.821	0.85	0.73	0.79	1.06	0.86	0.75	0.53	0.41	0.235	0.87	0.83

Table 5 shows the crude population size of the student with high-risk behaviors that were corrected by visibility factor in Table 6. The population size of the students who had extramarital heterosexual intercourse 18119.18 (56.55%) and consumed alcohol 10586.78 (33.04%) was significantly higher than that of the other groups, but the population size of the students who used opium was smaller than that of the rest of the groups 889.70 (2.77%). The results showed in detail in Table 6.

**Table 5:** The estimated population size of students with high-risk behaviors with a 95% confidence interval.

Target Group	Crude Estimate of the Frequency of Uncertain Cases	95% Confidence Interval		Crude Estimate of the Frequency of Certain Cases	95% Confidence Interval	
		Lower Bound	Upper Bound		Lower Bound	Upper Bound
Tramadol Use	2.49%	2.33%	2.68%	2.48%	2.30%	2.70%
Alcohol Consumption	14.40%	14%	14.80%	14.22%	13.84%	14.60%
Opium Use	0.63%	0.54%	0.71%	0.63%	0.54%	0.71%
Cannabis Use	1.39%	1.27%	1.52%	1.39%	1.27%	1.52%%
Extramarital Heterosexual Intercourse	13.33%	12.96%	13.71%	12.84%	12.48%	13.21%
Heterosexual Intercourse in Return for Money	3.49%	3.29%	3.70%	3.45%	3.25%	3.65%

**Table 6:** Estimates of the adjusted population size using the visibility factor.

Target Group	Adjusted Frequency Estimate %	95% Confidence Interval	
		Lower Bound	Upper Bound
Tramadol Use	2011.07 (6.27%)	2000.09 (6.01%)	2022.06 (6.54%)
Alcohol Consumption	10586.78 (33.04%)	10576.70 (32.53%)	10596.89 (33.56%)
Opium Use	889.70 (2.77%)	870.52 (2.59%)	908.92 (2.95%)
Cannabis Use	1543.66 (4.81%)	1528.67 (4.58%)	1558.65 (5.05%)
Extramarital Heterosexual Intercourse	18119.18 (56.55%)	18100.08 (56.01%)	18138.30 (57.10%)
Heterosexual Intercourse in Return for Money	3959.04 (12.35%)	3943.49 (11.99%)	3974.63 (12.71%)

## Discussion

According to the review of literature, the present research is one of the first studies to estimate the population size of students with HIV-related high-risk behaviors in Tehran and Alborz medical universities through the indirect method of NSU. Mean network size of participants was 8.14 (95%CI: 7.54, 8.75). According to the results of the multiple linear regression model network size of the participants was significantly different based on some socio-demographic characteristics.The students who were female, lived in home and studied at master or PhD degree significantly had less network size than who were male, lived in dormitory and studied at bachelor degree. These findings that seems logical, considering some cultural, social and values in Iranan society, is consistent with other studies [10, 22]. For example the male student due to their personal and social characteristics have more social relationship with other people than female. Also the students who live in dormitory and studied at bachelor degree have more opportunity and, also more free time to develop their relationships with other students to be socialize with them. Therefore it seems that these socio-demographic characteristics increase mean of students’ network size and it is more likely that students with large network size know more people who have risk behaviors.

According to the results of Table 3, in participants’ social network of close friends, the highest frequency pertained to those with extramarital heterosexual intercourse (5.53%) and the alcohol consumers (4.92%), which is consistent with the results obtained by Shokouhi et al. (2012) who also by using NSU method found that the consumption of alcohol is more frequent high risk behavior among general population especially in male in Kerman [1]. Although some studies that examined the prevalence of high-risk behaviors among university and high school students in Iran had yielded similar results, none of them have used NSU as an indirect method of population size estimation. According to the results of the multiple logistic regression some socio-demographic characteristics including age, gender, marital status, type of residence and academic degree had significant relationships with the likelihood of having close friends with certain high-risk behaviors. For example male significantly more likely than female knew close friends who had high-risk behaviors including tramadol use (AOR = 0.15, p<0.01), Alcohol consumption (AOR = 0.19, p<0.02), having extramarital heterosexual intercourse (AOR = 0.45, p<0.001), and heterosexual intercourse in return for money(AOR = 23.7, p<0.001). Although these differences seem understandable considering the nature of these subpopulations and the Iranian culture, this interesting finding highlight some barriers as age, gender, marital status, type of residence and academic degree that can effect on knowing friends with high risk behaviors. For example about barrier effect of “academic degree”, those in bachelor were more likely than others know friends with high risk behaviors. This is probably because they have relatively more free time to spend with their friends and other students. According to the findings of multiple regression findings in Table 2, male and female had different network size, and the identified groups that could be used for the estimates of these two groups also can be differed by gender. This finding concurs with the results obtained in a study at Ilam University that found gender to be involved in risk-taking behaviors among students[23]. Moreover, men have a greater freedom in the community and the family in most societies and are therefore more in contact with people with high-risk behaviors and are more exposed to these behaviors themselves [24].

The NSU results showed that the frequency of alcohol consumption and extramarital hetrosexual sex were higher than other risky behaviors. This finding is consistent with the results of a study conducted to estimate the population size of college student with high-risk behaviors using NSU in Kerman [25]. Sheikhzadeh et al. (2014) also reported alcohol consumption and extramarital heterosexual intercourse as the most common high-risk behaviors among university students[26]. In a study conducted by Zarrabi et al. (2009) at one of University of Medical Sciences in north of Iran, alcohol consumption (17.04%) was the most frequent high-risk behavior following smoking (26.36%) [27], and according to the results of a survey conducted in 2003 among opium-using students from public universities in Iran, alcohol consumption was the most frequent high-risk behavior and 20% of the students had consumed alcohol at least once in their life [28].In another study, Karami et al. found that the attitude toward extramarital sex is less strict among people who belong to a social network[29]. In the study by Emel Ege et al. (2011), 17.7% of university students had extramarital sexual intercourse, and these behaviors were significantly more common in the male than the female students[30]. In studies conducted in India, Nigeria and the US, extramarital sex was reported as a very frequent issue. Economic inequalities, the desire for modern living, sexual inequalities, morality problems, socioeconomic status, family characteristics and the influence of peers and the media are some of the factors contributing to the high frequency of these relations [29, 31–34]. The prevalence of sexual relations is significantly lower among Iranian students compared to students in other countries, which could be due to socio-cultural factors and the religious conditions dominating the country. In Muslim countries such as Iran, sex before marriage and extramarital sex are prohibited by religious laws and the government and are considered a legal crime [35].

Because of their higher sense of accountability, medical students experience greater stress than other students, and this pressure could be a reason for their greater inclination to drugs. Furthermore, students who share homes with each other are more likely to belong to social networks of people with high-risk behaviors and drug abuse compared to those who live with their families [27].

The present study used NSU to examine participants' social networks of HIV-related high-risk behaviors, including groups of drug users and those engaging in extramarital heterosexual intercourse; NSU provides one of the best data collection methods in such clandestine hard-to-count populations. Estimating the actual size of these populations is almost impossible with direct methods and there are no formal statistics on their actual size [11, 21].

In summary, the results showed that alcohol consumption and extramarital heterosexual intercourse are very common among the students. A local audience interested in the high-risk behaviors of Iranian youth may find these findings helpful, especially in terms of health policy-making and the design of special HIV prevention programs.

## Limitations

Despite providing a specific and accurate definition of "close friend", considering that the data were collected in self-report form, the estimates obtained cannot be flawless or perfectly accurate. Also, the results must be interpreted with more caution given certain limitation of the sampling that can effect on our point estimates, statistical tests, and confidence intervals. Moreover, given that this study was conducted by convenience sampling, the results cannot be representative of all student population of the country and generally all youth. Therefore the results only can be generalized to the students of medical university in Tehran and Karaj cities.

## Supporting information

S1 File (SAV)

S2 File (PDF)

S1 Questionnaire (PDF)
