# Supplementary material for: An indirect estimation of the population size of students with high-risk behaviors in select universities of medical sciences: A network scale-up study
Source: PLoS One. 2018 May 8;13(5):e0195364. doi: 10.1371/journal.pone.0195364 (PMC5940232; doi:10.1371/journal.pone.0195364)
Supplement: S2 File — (PDF) [file pone.0195364.s002.pdf]

دوستی نزدیک که عبارت است از: افرادی که به مدت حداقل ۶ ماه همدیگر را می شناسند و در دوهفته اخیر حداقل یک وعده غذا با هم خورده اند و ۲ ساعت نیز باهم وقت گذرانده اند.

سوالات مربوط به سو مصرف مواد و الکل و مسایل مربوط به آن

**ماده مصرفی**							
**-مصرف کننده ترامادول**			**ب:ازدیدگاه شما مصرف ترامادول درجامعه دانشجویی به چه میزان مورد قبول است؟**<br>**کم** ☐ **متوسط** ☐ **زیاد** ☐				
الف : آیا دوست دانشجویی در دانشگاههای علوم پزشکی تهران/البرز می شناسید که در شش ماه اخیر حداقل یکبار داروای ترامادول را بدون تجویز پزشک مصرف کرده باشد، چه برای تفنن و چه صورت مستمر	بلی ☐	خیر ☐					
	**ج: اگر بله لطفا تعداد را در زیر گروههای زیر بیان فرمایید:**		گاهی<br>مستمر	زیر ۲۲ سال<br>۲۵-۲۳سال<br>۳۰-۲۶ سال<br>بالای ۳۰ سال		کارشناسی<br>کارشناسی ارشد ☐<br>PhD ☐<br>دکتری عمومی ☐	**خوابگاهی** ☐<br>**غیر خوابگاهی:**<br>**خانه مستقل(مجردی)** ☐<br>**خانه گروهی(مجردی)** ☐<br>**با خانواده** ☐
	قطعی	مرد<br>زن					

ماده مصرفی		ب:ازدیدگاه شما مصرف الکل درجامعه دانشجویی به چه میزان مورد قبول است؟<br>کم ☐ متوسط ☐ زیاد ☐
مصرف کننده الکل	بلی ☐ خیر ☐	

الف: آیا دوست دانشجویی در دانشگاههای علوم پزشکی تهران/البرز می شناسید که در شش ماه اخیر حداقل یکبار مواد الکلی (مثل الکل، ودکا، ویسکی، شراب، آبجو و ...) رامصرف کرده باشد، چه برای تفنن و چه بصورت مستمر	ج:اگر بله لطفا تعداد را در زیر گروههای زیر بیان فرمایید:						
	قطعی ☐	مرد ☐<br>زن ☐	گاهی ☐<br>مستمر	زیر ۲۲ سال<br>۲۵-۲۳سال<br>۳۰-۲۶ سال<br>بالای ۳۰ سال	☐<br>☐<br>☐<br>☐	کارشناسی ☐<br>کارشناسی ارشد ☐<br>PhD ☐<br>دکتری عمومی ☐	**خوابگاهی** ☐<br>**غیر خوابگاهی:** ☐<br>**خانه مستقل(مجردی)** ☐<br>**خانه گروهی(مجردی)** ☐<br>**با خانواده** ☐

مصرف کننده تریاک			ب:ازدیدگاه شما مصرف تریاک درجامعه دانشجویی به چه میزان مورد قبول است؟<br>کم ☐ متوسط ☐ زیاد ☐
الف : آیا دوست دانشجویی در دانشگاههای علوم پزشکی تهران/البرز می شناسید که در شش ماه اخیر حداقل یکبار تریاک استفاده کرده باشد چه برای تفنن و چه به دلیل استفاده مستمر	بلی ☐	خیر ☐	

	ج: اگر بله لطفا تعداد را در زیر گروههای زیر بیان فرمایید:						
	قطعی	مرد ☐<br>زن ☐	گاهی ☐<br>مستمر ☐	زیر ۲۲ سال<br>۲۵-۲۳سال<br>۳۰-۲۶ سال<br>بالای ۳۰ سال	☐<br>☐<br>☐<br>☐	کارشناسی ☐<br>کارشناسی ارشد ☐<br>PhD ☐<br>دکتری عمومی ☐	**خوابگاهی** ☐<br>**غیر خوابگاهی:**<br>**خانه مستقل(مجردی)** ☐<br>**خانه گروهی(مجردی)** ☐<br>**با خانواده** ☐

**ماده مصرفی**			**ب:ازدیدگاه شما مصرف مواد روانگردان درجامعه دانشجویی به چه میزان مورد قبول است؟**<br>**کم** ☐ **متوسط** ☐ **زیاد** ☐				
**مصرف کننده مواد روانگردان**	بلی ☐	**خیر** ☐					
الف: آیا دوست دانشجویی در دانشگاههای علوم پزشکی تهران/البرز می شناسید که در <u>شش ماه اخیر</u> حداقل یکبار **LSD** آمفتامین ، اکستازی رامصرف کرده باشد، چه برای تفنن و چه بصورت مستمر	**ج:اگر بله لطفا تعداد را در زیر گروههای زیر بیان فرمایید:**<br>قطعی ☐	مرد ☐<br>زن ☐	گاهی ☐<br>مستمر	زیر ۲۲ سال<br>۲۵-۲۳سال<br>۳۰-۲۶ سال<br>بالای ۳۰ سال	☐<br>☐<br>☐<br>☐	کارشناسی ☐<br>کارشناسی ارشد ☐<br>PhD ☐<br>دکتری عمومی ☐	**خوابگاهی** ☐<br>**غیر خوابگاهی:** ☐<br>**خانه مستقل(مجردی)** ☐<br>**خانه گروهی(مجردی)** ☐<br>**با خانواده**
**مصرف کننده شیشه/کرک**			**ب:ازدیدگاه شما مصرف شیشه/کرک درجامعه دانشجویی به چه میزان مورد قبول است؟**<br>**کم** ☐ **متوسط** ☐ **زیاد** ☐				
	بلی ☐	خیر ☐					
الف : آیا دوست دانشجویی در دانشگاههای علوم پزشکی تهران/البرز می شناسید که در <u>شش ماه اخیر</u> حداقل یکبار شیشه/کرک استفاده کرده باشد چه برای تفنن و چه به دلیل استفاده مستمر	**ج: اگر بله لطفا تعداد را در زیر گروههای زیر بیان فرمایید:**<br>قطعی ☐	مرد ☐<br>زن ☐	گاهی ☐<br>مستمر ☐	زیر ۲۲ سال<br>۲۵-۲۳سال<br>۳۰-۲۶ سال<br>بالای ۳۰ سال	☐<br>☐<br>☐<br>☐	کارشناسی ☐<br>کارشناسی ارشد ☐<br>PhD ☐<br>دکتری عمومی ☐	**خوابگاهی** ☐<br>**غیر خوابگاهی:**<br>**خانه مستقل(مجردی)** ☐<br>**خانه گروهی(مجردی)** ☐<br>**با خانواده** ☐

ماده مصرفی			ب:ازدیدگاه شما تحت درمان با متادون درجامعه دانشجویی به چه میزان مورد قبول است؟<br>کم ☐ متوسط ☐ زیاد ☐				
تحت درمان با متادون	بلی ☐	خیر ☐					
الف :آیا دوست دانشجویی در دانشگاههای علوم پزشکی تهران/البرز می شناسید که در شش ماه اخیر تحت درمان با متادون باشد.	قطعی ☐	ج:اگر بله لطفا تعداد را در زیر گروههای زیر بیان فرمایید:<br>مرد ☐<br>زن ☐	گاهی ☐<br>مستمر ☐	زیر ۲۲ سال<br>۲۵-۲۳سال<br>۳۰-۲۶ سال<br>بالای ۳۰ سال	☐<br>☐<br>☐<br>☐	کارشناسی ☐<br>کارشناسی ارشد ☐<br>PhD ☐<br>دکتری عمومی ☐	خوابگاهی ☐<br>غیر خوابگاهی:<br>خانه مستقل(مجردی) ☐<br>خانه گروهی(مجردی) ☐<br>با خانواده ☐
مصرف کننده مصرف کننده مواد به روش تزریق			ب:ازدیدگاه شما مصرف مواد به روش تزریق درجامعه دانشجویی به چه میزان مورد قبول است؟<br>کم ☐ متوسط ☐ زیاد ☐				
الف : آیا دوست دانشجویی در دانشگاههای علوم پزشکی تهران/البرز می شناسید که در شش ماه اخیر حداقل یکبار مواد به روش تزریق چه برای تفنن و چه بصورت مستمر استفاده کرده باشد .	بلی ☐<br>قطعی ☐	خیر ☐<br>ج: اگر بله لطفا تعداد را در زیر گروههای زیر بیان فرمایید:<br>مرد ☐<br>زن ☐	گاهی ☐<br>مستمر ☐	زیر ۲۲ سال<br>۲۵-۲۳سال<br>۳۰-۲۶ سال<br>بالای ۳۰ سال	☐<br>☐<br>☐<br>☐	کارشناسی ☐<br>کارشناسی ارشد ☐<br>PhD ☐<br>دکتری عمومی ☐	خوابگاهی ☐<br>غیر خوابگاهی:<br>خانه مستقل(مجردی) ☐<br>خانه گروهی(مجردی) ☐<br>با خانواده ☐

سوالات مربوط به ارتباطات جنسی و مسایل مربوط به آن

<table dir="rtl">
<tr><td>ارتباطات جنسی</td><td colspan="2"></td><td colspan="5">ب:ازدیدگاه شما ارتباط جنسی با جنس مخالف در ازاء پرداخت پول یا تقبل هر گونه هزینه دیگر مالی و یا غیر مالی درجامعه دانشجویی به چه میزان مورد قبول است؟
کم ☐ متوسط ☐ زیاد ☐</td></tr>
<tr><td rowspan="3">- ارتباط جنسی با جنس مخالف در ازای پرداخت پول و یا تقبل هر گونه هزینه دیگر مالی و یا غیر مالی</td><td>بلی ☐</td><td>خیر ☐</td><td colspan="5"></td></tr>
<tr><td colspan="2">ج:اگر بله لطفا تعداد را در زیر گروههای زیر بیان فرمایید:</td><td rowspan="3">گاهی ☐
مستمر ☐</td><td rowspan="3">زیر ۲۲ سال
۲۳-۲۵سال
۲۶-۳۰ سال
بالای ۳۰ سال</td><td rowspan="3">☐
☐
☐
☐</td><td rowspan="3">کارشناسی ☐
کارشناسی ارشد ☐
PhD ☐
دکتری عمومی ☐</td><td rowspan="3">خوابگاهی ☐
غیر خوابگاهی:
خانه مستقل(مجردی) ☐
خانه گروهی(مجردی) ☐
با خانواده ☐</td></tr>
<tr><td rowspan="2">قطعی ☐</td><td rowspan="2">مرد ☐
زن ☐</td></tr>
<tr><td>الف : آیا دوست دانشجویی در دانشگاههای علوم پزشکی تهران/البرز می شناسید که در شش ماه گذشته حداقل با یک فرد در ازای پرداخت پول و یا تقبل هر گونه هزینه دیگر مالی و یا غیر مالی ارتباط جنسی داشته است.</td></tr>
<tr><td>- ارتباط جنسی با جنس مخالف در خارج از چارچوب ازدواج</td><td></td><td></td><td colspan="5">ب:ازدیدگاه شما ارتباط جنسی با جنس مخالف در خارج از چارچوب ازدواج درجامعه دانشجویی به چه میزان مورد قبول است؟ کم ☐ متوسط ☐ زیاد ☐</td></tr>
<tr><td rowspan="3">الف : آیا دوست دانشجویی در دانشگاههای علوم پزشکی تهران/البرز می شناسید که در شش ماه اخیر حداقل با یک فرد در خارج از چارچوب ازدواج ارتباط جنسی داشته است(بدون پرداخت پول و یا تقبل هر گونه هزینه دیگر مالی و یا غیر مالی)</td><td>بلی ☐</td><td>خیر ☐</td><td colspan="5"></td></tr>
<tr><td colspan="2">ج: اگر بله لطفا تعداد را در زیر گروههای زیر بیان فرمایید:</td><td rowspan="2">گاهی ☐
مستمر ☐</td><td rowspan="2">زیر ۲۲ سال
۲۳-۲۵سال
۲۶-۳۰ سال
بالای ۳۰ سال</td><td rowspan="2">☐
☐
☐
☐</td><td rowspan="2">کارشناسی ☐
کارشناسی ارشد ☐
PhD ☐
دکتری عمومی ☐</td><td rowspan="2">خوابگاهی ☐
غیر خوابگاهی:
خانه مستقل(مجردی) ☐
خانه گروهی(مجردی) ☐
با خانواده ☐</td></tr>
<tr><td>قطعی ☐</td><td>مرد ☐
زن ☐</td></tr>
</table>

سوالات مربوط به ارتباطات جنسی و مسایل مربوط به آن

<table>
<tr><td colspan="2">**ارتباطات جنسی**</td><td colspan="2"></td><td colspan="5">**ب:ازدیدگاه شما ارتباط جنسی با هم جنس در ازای پول درجامعه دانشجویی به چه میزان مورد قبول است؟**
کم ☐ **متوسط** ☐ **زیاد** ☐</td></tr>
<tr><td rowspan="2">**دوست دانشجوی که در ازاء پول با هم جنس ارتباط جنسی داشته باشد.**</td><td>بلی ☐</td><td>**خیر** ☐</td><td colspan="5"></td></tr>
<tr><td colspan="2">**ج:اگر بله لطفا تعداد را در زیر گروههای زیر بیان فرمایید:**</td><td rowspan="2">گاهی ☐
مستمر ☐</td><td rowspan="2">زیر ۲۲ سال
۲۵-۲۳سال
۳۰-۲۶ سال
بالای ۳۰ سال</td><td rowspan="2">☐
☐
☐
☐</td><td rowspan="2">کارشناسی ☐
کارشناسی ارشد ☐
PhD ☐
دکتری عمومی ☐</td><td rowspan="2">**خوابگاهی** ☐
غیر خوابگاهی: ☐
خانه مستقل(مجردی) ☐
خانه گروهی(مجردی) ☐
با خانواده ☐</td></tr>
<tr><td>**الف** : آیا دوست دانشجویی در دانشگاههای علوم پزشکی تهران/البرز می شناسید که در شش ماه گذشته با هم جنس خود در ازاء پول ارتباط جنسی داشته باشد.</td><td>قطعی ☐</td><td>مرد ☐
زن ☐</td></tr>
<tr><td>**مصرف مواد قبل یا حین روابط جنسی**</td><td></td><td></td><td colspan="5">**ب:ازدیدگاه شما مصرف مواد قبل یا حین روابط جنسی درجامعه دانشجویی به چه میزان مورد قبول است؟**
کم ☐ **متوسط** ☐ **زیاد** ☐</td></tr>
<tr><td rowspan="3">**الف** : آیا دوست دانشجویی در دانشگاههای علوم پزشکی تهران/البرز می شناسید که در شش ماه اخیر حداقل یکبار قبل یا حین برقراری روابط جنسی مواد مصرف کرده باشد.</td><td>بلی ☐</td><td>خیر ☐</td><td colspan="5"></td></tr>
<tr><td colspan="2">**ج: اگر بله لطفا تعداد را در زیر گروههای زیر بیان فرمایید:**</td><td rowspan="2">گاهی ☐
مستمر ☐</td><td rowspan="2">زیر ۲۲ سال
۲۵-۲۳سال
۳۰-۲۶ سال
بالای ۳۰ سال</td><td rowspan="2">☐
☐
☐
☐</td><td rowspan="2">کارشناسی ☐
کارشناسی ارشد ☐
PhD ☐
دکتری عمومی ☐</td><td rowspan="2">**خوابگاهی** ☐
غیر خوابگاهی:
خانه مستقل(مجردی) ☐
خانه گروهی(مجردی) ☐
با خانواده ☐</td></tr>
<tr><td>قطعی ☐</td><td>مرد ☐
زن ☐</td></tr>
</table>

لطفاً جهت تحلیل بهتر نتایج به سوالات زیر پاسخ دهید:

سن: **شغل:** **جنس: مرد** ☐ **زن** ☐

وضعیت تاهل: مجرد ☐ **متاهل** ☐ **مطلقه/همسر فوت کرده** ☐

مقطه تحصیلی: کارشناس ☐ **کارشناسی ارشد** ☐ **دکتری** ☐ **PhD** ☐

محل سکونت: خوابگاهی ☐ **زندگی با خانواده** ☐ **خانه مستقل(مجردی)** ☐ **خانه گروهی(مجردی)** ☐
