# Supplementary material for: An indirect estimation of the population size of students with high-risk behaviors in select universities of medical sciences: A network scale-up study
Source: PLoS One. 2018 May 8;13(5):e0195364. doi: 10.1371/journal.pone.0195364 (PMC5940232; doi:10.1371/journal.pone.0195364)
Supplement: S1 Questionnaire — (PDF) [file pone.0195364.s003.pdf]

Close friends mean people who have known each other for at least six months, and have had at least one meal together in the last two weeks and spent two hours with each other.

<table>
<tr><td>Substance</td><td colspan="2"></td><td colspan="5"></td></tr>
<tr><td>Tramadol user</td><td></td><td></td><td colspan="5">In your view, how acceptable is tramadol use in the student community?</td></tr>
<tr><td rowspan="3">Do you have a student friend in Tehran or Alborz universities of medical sciences who has used tramadol at least once in the last six months without doctor's prescription for fun or continuously</td><td>Yes</td><td>No</td><td colspan="5">Low Medium High</td></tr>
<tr><td colspan="2">If yes, please state quantity under the groups below</td><td rowspan="2">__________
Occasionally
Continuously</td><td></td><td></td><td></td><td></td></tr>
<tr><td>Definitely</td><td>Male
Female</td><td>Under 22
23-25
26-30
Older than 30</td><td></td><td>Bachelor's degree
Master's degree
PhD
General physician</td><td>Dormitory:
Non-dorm
Personal home (bachelor's)
Group (shared) house (bachelor's)
Family home</td></tr>
</table>

Questions about substance and alcohol abuse and related issues

Substance							
Alcohol user			In your view, how acceptable is alcohol use in the student community?				
Do you have a student friend in Tehran or Alborz universities of medical sciences who has used alcoholic substances (alcohol, vodka, whiskey, beer,…) at least once in the last six months for fun or continuously	Yes	No	Low Medium High				
	If yes, please state quantity under the groups below		__________				
	Definitely	Male<br>Female	Occasionally<br>Continuously	Under 22<br>23-25<br>26-30<br>Older than 30		Bachelor's degree<br>Master's degree<br>PhD<br>General physician	Dormitory:<br>Non-dorm<br>Personal home (bachelor's)<br>Group (shared) house (bachelor's)<br>Family home

Substance							
Opium user			In your view, how acceptable is opium use in the student community?				
Do you have a student friend in Tehran or Alborz universities of medical sciences who has used opium at least once in the last six months for fun or continuously	Yes	No	Low Medium High				
	If yes, please state quantity under the groups below		__________				
	Definitely	Male<br>Female	Occasionally<br>Continuously	Under 22<br>23-25<br>26-30<br>Older than 30		Bachelor's degree<br>Master's degree<br>PhD<br>General physician	Dormitory:<br>Non-dorm<br>Personal home (bachelor's)<br>Group (shared) house (bachelor's)<br>Family home

<table>
<tr><td>Substance</td><td colspan="2"></td><td colspan="5"></td></tr>
<tr><td>Psychotropic medication user</td><td></td><td></td><td colspan="5">In your view, how acceptable is psychotropic medication use in the student community?</td></tr>
<tr><td rowspan="3">Do you have a student friend in Tehran or Alborz universities of medical sciences who has used LSD, amphetamine, ecstasy at least once in the last six months for fun or continuously</td><td>Yes</td><td>No</td><td colspan="5">Low Medium High</td></tr>
<tr><td colspan="2">If yes, please state quantity under the groups below</td><td rowspan="2">__________
Occasionally
Continuously</td><td></td><td></td><td></td><td></td></tr>
<tr><td>Definitely</td><td>Male
Female</td><td>Under 22
23-25
26-30
Older than 30</td><td></td><td>Bachelor's degree
Master's degree
PhD
General physician</td><td>Dormitory:
Non-dorm
Personal home (bachelor's)
Group (shared) house (bachelor's)
Family home</td></tr>
</table>

<table>
<tr><td>Substance</td><td colspan="2"></td><td colspan="5"></td></tr>
<tr><td>Crystal/crack user</td><td></td><td></td><td colspan="5">In your view, how acceptable is crystal/crack use in the student community?</td></tr>
<tr><td rowspan="3">Do you have a student friend in Tehran or Alborz universities of medical sciences who has used crystal/crack at least once in the last six months for fun or continuously</td><td>Yes</td><td>No</td><td colspan="5">Low Medium High</td></tr>
<tr><td colspan="2">If yes, please state quantity under the groups below</td><td rowspan="2">__________
Occasionally
Continuously</td><td></td><td></td><td></td><td></td></tr>
<tr><td>Definitely</td><td>Male
Female</td><td>Under 22
23-25
26-30
Older than 30</td><td></td><td>Bachelor's degree
Master's degree
PhD
General physician</td><td>Dormitory:
Non-dorm
Personal home (bachelor's)
Group (shared) house (bachelor's)
Family home</td></tr>
</table>

Substance							
Methadone therapy			In your view, how acceptable is receiving methadone therapy in the student community?				
Do you have a student friend in Tehran or Alborz universities of medical sciences who has been receiving methadone therapy in the last six months	Yes	No	Low Medium High				
	If yes, please state quantity under the groups below		__________				
	Definitely	Male<br>Female	Occasionally<br>Continuously	Under 22<br>23-25<br>26-30<br>Older than 30		Bachelor's degree<br>Master's degree<br>PhD<br>General physician	Dormitory:<br>Non-dorm<br>Personal home (bachelor's)<br>Group (shared) house (bachelor's)<br>Family home

Substance							
Injection drug user			In your view, how acceptable is injection drug use in the student community?				
Do you have a student friend in Tehran or Alborz universities of medical sciences who has used injection drugs at least once in the last six months for fun or continuously	Yes	No	Low Medium High				
	If yes, please state quantity under the groups below		__________				
	Definitely	Male<br>Female	Occasionally<br>Continuously	Under 22<br>23-25<br>26-30<br>Older than 30		Bachelor's degree<br>Master's degree<br>PhD<br>General physician	Dormitory:<br>Non-dorm<br>Personal home (bachelor's)<br>Group (shared) house (bachelor's)<br>Family home

Questions about sexual relationships and associated problems

Sexual relationships			In your view, how acceptable is sexual relationship with the opposite sex for money or any other financial or non-financial favors in the student community?				
Sexual relationship with the opposite sex for money or any other financial or non-financial favors	Yes	No	Low	Medium		High	
	If yes, please state quantity under the groups below		__________				
Do you have a student friend in Tehran or Alborz universities of medical sciences who has had sexual relationship with at least one person in the last six months for money or any other financial or non-financial favors	Definitely	Male<br>Female	Occasionally<br>Continuously	Under 22<br>23-25<br>26-30<br>Older than 30		Bachelor's degree<br>Master's degree<br>PhD<br>General physician	Dormitory:<br>Non-dorm<br>Personal home (bachelor's)<br>Group (shared) house (bachelor's)<br>Family home

Sexual relationships with the opposite sex outside marriage			In your view, how acceptable is sexual relationship with the opposite sex outside marriage in the student community?				
Do you have a student friend in Tehran or Alborz universities of medical sciences who has had sex outside marriage with at least one person in the last six months (without payment of money or any other financial or non-financial favors)	Yes	No	Low	Medium		High	
	If yes, please state quantity under the groups below		___________				
	Definitely	Male<br>Female	Occasionally<br>Continuously	Under 22<br>23-25<br>26-30<br>Older than 30		Bachelor's degree<br>Master's degree<br>PhD<br>General physician	Dormitory:<br>Non-dorm<br>Personal home (bachelor's)<br>Group (shared) house (bachelor's)<br>Family home

Questions about sexual relationships and associated problems

Sexual relationships			In your view, how acceptable is sexual relationship with the same sex for money in the student community?				
A student friend who has had sexual relationship with the same sex for money<br>____________<br>Do you have a student friend in Tehran or Alborz universities of medical sciences who has had sexual relationship with the same sex in the last six months for money	Yes	No	Low Medium High				
	If yes, please state quantity under the groups below		__________				
	Definitely	Male<br>Female	Occasionally<br>Continuously	Under 22<br>23-25<br>26-30<br>Older than 30		Bachelor's degree<br>Master's degree<br>PhD<br>General physician	Dormitory:<br>Non-dorm<br>Personal home (bachelor's)<br>Group (shared) house (bachelor's)<br>Family home

Drug use before or during sex			In your view, how acceptable is drug use before or during sexual relationship in the student community?				
Do you have a student friend in Tehran or Alborz universities of medical sciences who has used drugs before or during sex at least once in the last six months	Yes	No	Low Medium High				
	If yes, please state quantity under the groups below		__________				
	Definitely	Male<br>Female	Occasionally<br>Continuously	Under 22<br>23-25<br>26-30<br>Older than 30		Bachelor's degree<br>Master's degree<br>PhD<br>General physician	Dormitory:<br>Non-dorm<br>Personal home (bachelor's)<br>Group (shared) house (bachelor's)<br>Family home

Please answer the following questions to enable better analysis of the results

Age:

Occupation:

Gender: male: female:

Marital status: single: married: divorced/widowed:

Education qualification: bachelor's degree: Master's degree: Doctorate: PhD:

Place of residence: Dormitory: living with family: bachelor's house (singles): group house: shared (singles)
